# A Comparative Study of National Family Health Survey-4 and National Family Health Survey-5 of Nutritional Indicators in Chhattisgarh

**DOI:** 10.7759/cureus.55524

**Published:** 2024-03-04

**Authors:** Anupriya Jha, Aditi Chandrakar

**Affiliations:** 1 Community and Family Medicine, All India Institute of Medical Sciences, Raipur, IND

**Keywords:** overweight, underweight, wasting, stunting, chhattisgarh, nfhs, nutritional indicators

## Abstract

Globally, public health issues related to malnutrition exist. One of the countries grappling with challenges in combating anemia and malnutrition is India, including the state of Chhattisgarh. The National Family Health Survey-5 (NFHS-5) data show that the advancements made in the National Family Health Survey-4 (NFHS-4) were reversed in NFHS-5. Despite having several programs and policies in place, Chhattisgarh has not yet utilized all of its potential to demonstrate exponential reductions in anemia and malnutrition. This study highlights probable factors and inter-district variations to provide an overview of the nutritional condition of districts in Chhattisgarh compared to NFHS-4. Children under five who are severely wasted, stunted, or experiencing both conditions exhibit a lower prevalence. An immediate warning indication was the rise in anemia prevalence across all age categories. In Chhattisgarh, when comparing NFHS-5 to NFHS-4, the study identified a reduced frequency of direct determinants and an increased coverage of nutrition-specific treatments. The state of Chhattisgarh has seen a significant improvement in underlying factors, including the number of homes with power and drinking water quality. It also describes the shortcomings and advancements in the inter-district variations among the coverage factors. Instead of focusing on raising the nutritional indicators for Chhattisgarh, this study also includes initiatives made by states that have fared better in terms of those metrics.

## Introduction and background

A healthier population is greatly influenced by nutrition-related factors, but malnutrition, in all its forms, is a major risk factor that, over time, negatively affects morbidity. Since it raises the risk of illness and mortality as well as excessive medical costs, malnutrition has long been seen as a serious public health concern. Youngsters have unique and crucial nutritional needs [1]. Malnutrition is a chronic or subacute nutrition state that causes decreased body composition and decreased function. It includes under- or overnutrition and inflammatory activity within it [2]. Undernutrition, a form of malnutrition, occurs when a person consumes insufficient energy from their diet to meet their physiological demands [3]. The phrase *double burden of malnutrition* (DBM) refers to undernutrition paired with overweight or obesity in a culture [4]. Thus, the triple malnutrition burden is caused by a combination of undernutrition, overnutrition, and micronutrient deficiencies [5,6]. Its range is broad; difficulties with underweight, wasting, stunting, and micronutrient deficiencies are at one end, while obesity and overweight concerns are at the other [4]. Because undernutrition is linked to about half of all child fatalities under five, especially in low- and middle-income countries (LMICs), it continues to be a serious public health concern worldwide [7]. Out of all children under five, 22% are predicted to be stunted, with 150 million of them being under the age of five. Since LMICs bear the brunt of the stunting epidemic, 98% of stunted children reside there [8]. High rates of underweight among women in LMICs have long been a severe public health concern, just as it has been for adults. The epidemiological trend has, however, noticeably shifted from underweight to overweight as a result of rising urbanization and globalization in LMICs, as well as associated alterations in lifestyle and behavior [5,9,10]. Overweight and obesity are becoming more commonplace worldwide, particularly in urban regions and low-income nations [11]. Currently, 5·6% of under-five children, 17·5% of girls and young women (5-19 years old), and 39·2% of women (≥18 years old) worldwide are obese or overweight. Anemia has a significant impact on social, economic, and human health development, making it a major global public health concern. Anemia can cause physical and cognitive deficits, fatigue, and reduced productivity [6,12]. In the lack of micronutrient statistics, the prevalence of anemia, which stands for micronutrient deficiencies, is 43% among under-five children worldwide and 32·8% among reproductive-age women (15-49 years old) [6]. Indian children are among the world's most at-risk for stunting and wasting, underweight. Overnutrition in children has become more common across the country. The Mid-Day Meal (MDM) program for school-age children and the Integrated Child Development Services (ICDSs) program for preschoolers is the largest food supplementation program in the world that India is developing to address the high incidence of childhood undernutrition. These initiatives aim to help kids close their dietary energy deficit. To reach the SDG targets, there is a need to have programs in place that deal with the prevention, detection, and treatment of under and overnutrition in children [13]. Chhattisgarh is one of the Indian states struggling to overcome anemia and malnutrition. To give estimates of various indicators at the national, state/union territory (UT), and district levels throughout India is the purpose of the National Family Health Survey (NFHS). It is a multi-round, comprehensive survey of homes. NFHS-5 provides district-level estimates for several important metrics. The purpose of this report is to present comprehensive data on nutritional indicators, such as children's nutritional status, adults' nutritional status (age 15-49 years), anemia in children, adult males, reproductive age groups women, pregnant women, and districts in Chhattisgarh state that are considered as high prevalent.

## Review

Methodology

We compared the summary data of NFHS-5 and NFHS-4 with the state and district fact sheets of Chhattisgarh published by the International Institute for Population Sciences (IIPS). The assessment of global nutrition targets for maternal, infant, and early childhood nutrition involved the evaluation of various outcome indicators. These indicators encompassed the anemia status of women of reproductive age, as well as measures of stunting, wasting, and severe wasting in children. Several underlying and immediate nutrition determinants were also looked at. Nonetheless, we note how nutrition-specific interventions vary in terms of coverage throughout the life cycle. Data from NFHS-5 and NFHS-4 were used to prepare all the maps and figures.

Nutrition performance patterns in Chhattisgarh

**Figure 1:**
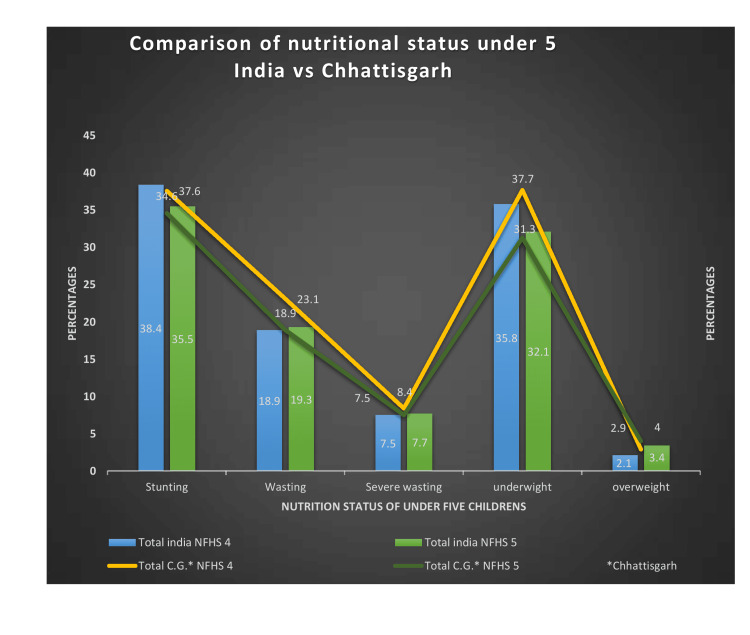
Nutritional status of children under five years. Sources: [16,18]. NFHS, National Family Health Survey

Figure 1 illustrates how, nationwide, the proportion of under-five children with stunting and underweight fell dramatically from NFHS-4 to NFHS-5. In India, 18.9% of under-five children were wasted, 38.4% were stunted, and 35.8% were underweight in 2015-2016. Compared to NFHS-4, data from NFHS-5 showed a lower prevalence: 35.5% of under-five children were stunted, 19.3% were wasting, and 32.1% were underweight. The severely wasted children’s prevalence in India rose from 7.5% to 7.7%, and 3.4% of children under five in India were overweight

The NFHS-5 survey conducted in Chhattisgarh state revealed a decrease in the percentage of stunted, underweight, severely wasted, and wasted children under five children. Underweight prevalence fell from 37.7% to 31.3% in NFHS-5, while severe wasting prevalence increased from 7.5% to 8.4%, and stunting slightly declined from 37.6% to 34.6%. While the number of under-five children who reported wasting decreased from 23.1% to 18.9%, the percentage of overweight children grew from 2.9% to 4%.

**Figure 2:**
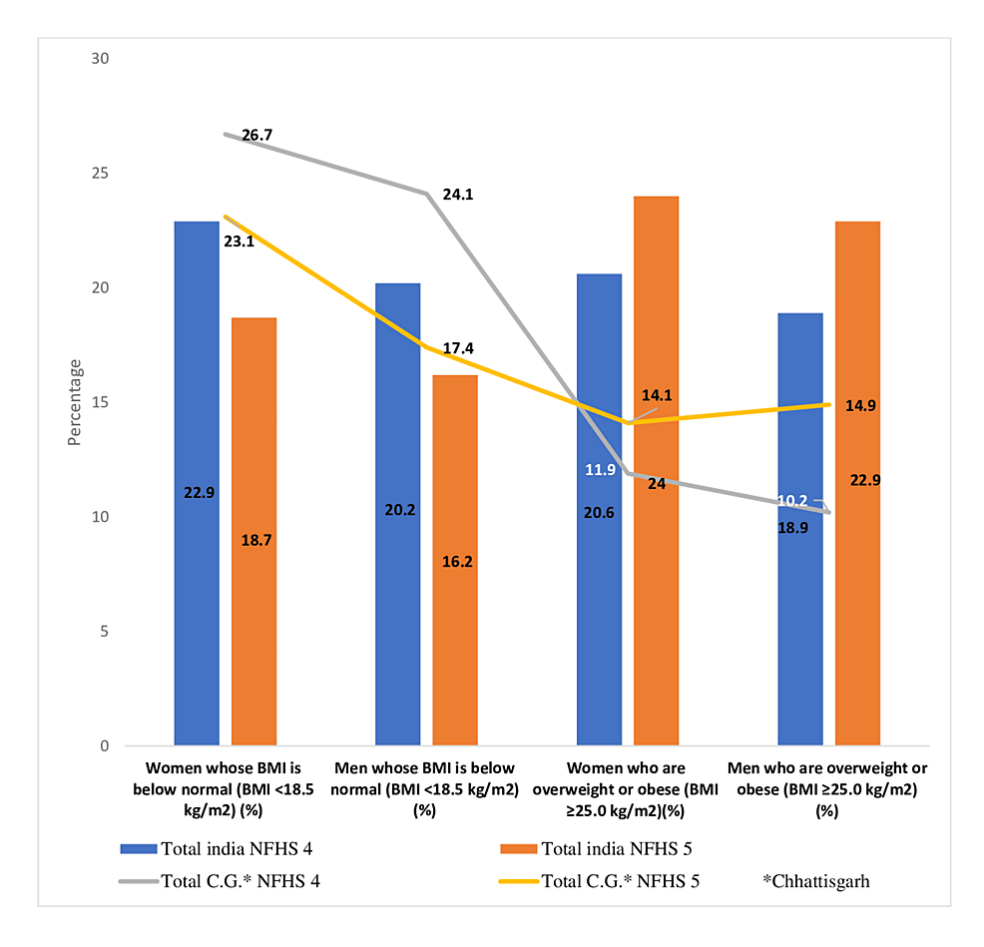
Nutritional Status of adults (age 15-49 years) Sources: [16,18]. NFHS, National Family Health Survey; BMI, body mass index

Now, when examining the adult nutrition status in India, we found that compared to men, the percentage of women with a BMI below normal (<18.5 kg/m^2^) decreased significantly from 22.9% to 18.7% nationwide, according to the comparative data between NFHS-4 and NFHS-5 shown in Figure 2. In India, there was an increase in the percentage of obese or overweight women (BMI ≥25 kg/m^2^) by 3.4%, and the percentage of obese or overweight males increased from 18.9% to 22.9%.

Contrastingly, Chhattisgarh state exhibited improved performance in the NFHS-5 survey, with a significant decrease in the prevalence of women and men with BMIs below normal (<18.5 kg/m^2^), from 26.7% and 24.1%, respectively. However, in comparison to NFHS-4, the percentages of overweight and obese women increased from 11.9% to 14.1%. Additionally, there was a notable increase in the prevalence of overweight and obese men, from 10.2% to 14.9%.

**Figure 3:**
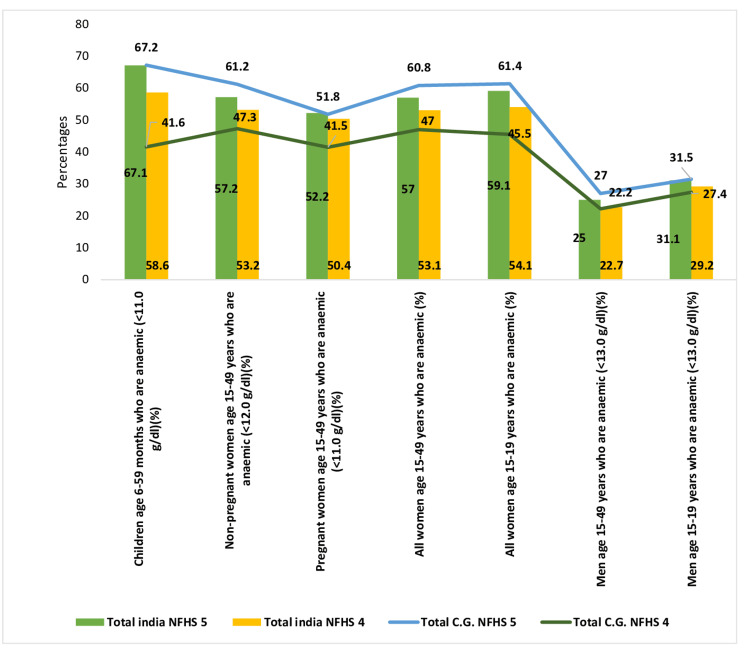
Prevalence of anemia. Sources: [16,18]. NFHS, National Family Health Survey

Figure 3 unveils that anemia is a major concern in Chhattisgarh state and that all age groups show a significant rise in the prevalence same as the nation’s status. In India, there has been an evident rise in anemia prevalence across various demographics when comparing data from both national surveys. Among 6- to 59-month-old children, the prevalence increased by 8.5%. Similarly, in 15- to 49-year-old non-pregnant women, there was a 4% increase, while in pregnant women of the same age group, the prevalence changed from 50.4% to 52.2%. Among all anemic women aged 15 to 49 years, it surged from 53.1% to 57%, and in anemic men of the same age group, it rose from 22.7% to 25%.

The trend toward increased anemia prevalence is also evident in Chhattisgarh state. In children aged 6 to 59 months, the prevalence escalated from 41.6% to 67.2%. Similarly, in 15- to 49-year-old non-pregnant women, the prevalence increased from 47.3% to 61.2%, and in pregnant women of the same age group, it rose from 41.5% to 51.8%. Among all anemic women aged 15 to 49 years, the prevalence surged from 47.0% to 60.8%, and in anemic men aged 15 to 49 years with hemoglobin levels <13 g/dL, there was an increase.

Highlights of Chhattisgarh

**Figure 4:**
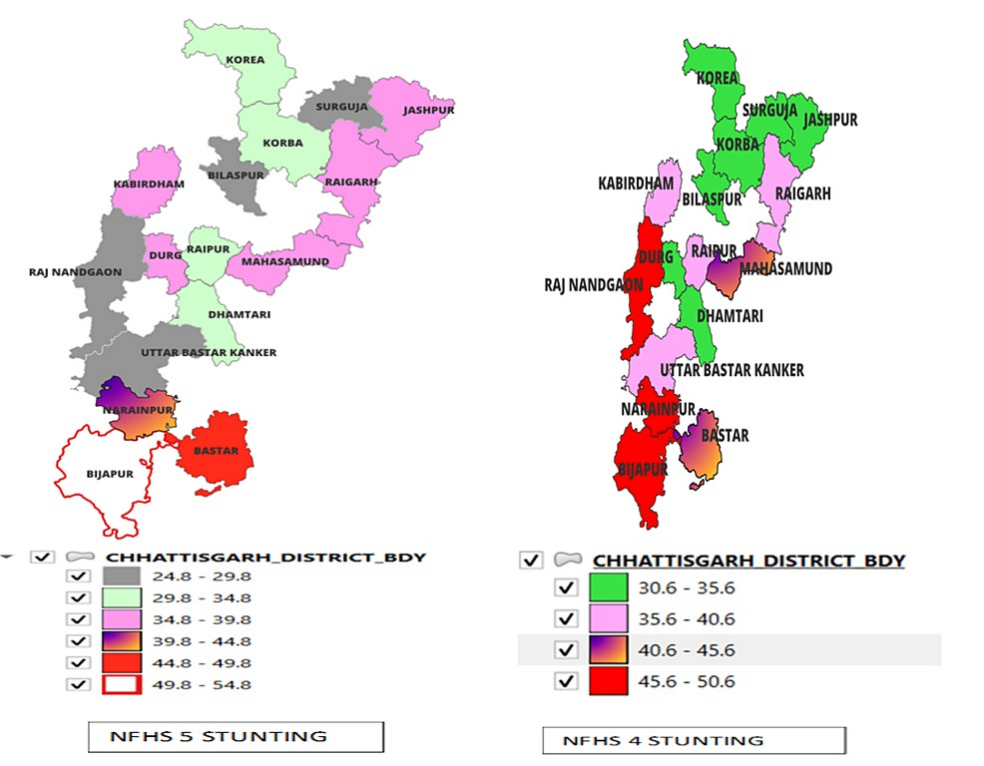
Comparison of stunting under-five children in NFHS-4 and NFHS-5 in the districts of Chhattisgarh. Sources: [16,18]. NFHS, National Family Health Survey

In NFHS-4, there were 18 districts, and in NFHS-5, the number increased to 27 because 9 districts were added for better clarity in 2012. So, during the comparison, only 18 districts were considered. Nine districts, namely Balod, Balod Bazar, Bemetara, Balarampur, Gariyaband, Kondagaon, Mungeli, Surjapur, and Sukma, were added in NFHS-5 only and therefore not included in the comparison in the map. (The following maps were prepared using QGIS software, wherein the Graduated option was utilized, and an equal interval was selected for classifying data. Therefore, the color coding only represents prevalence in percentage for presentation purposes.) The state percentage of stunted children under five (height for age) in NFHS-5 is lower than in NFHS-4. However, in Chhattisgarh state, as Figure 4 shows, 7 out of 18 districts have demonstrated a rise in the proportion of stunted (height for age) children under five. It includes Bastar, Bijapur, Dantewada, Durg, Jashpur, Korba, Koriya. Districts Rajnangao, Kaker, and Raigarh show a significant decrease in stunting in NFHS-5 (Appendices A-B).

**Figure 5:**
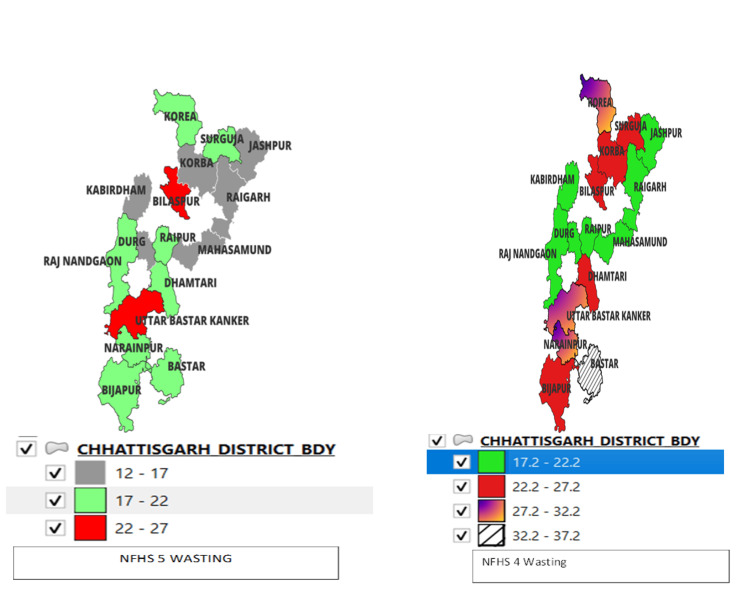
Comparison of wasting under-five children in NFHS-4 and NFHS-5 in the districts of Chhattisgarh. Sources: [16,18]. NFHS, National Family Health Survey

When comparing wasting in NFHS-4 and NFHS-5, Figure 5 shows that 4 out of 18 districts perform well with a decrease in the percentage of wasting. These districts are Koriya, Korba, Bastar, and Dantewada. In contrast, Janjgir-Champa, Rajnandgaon, and Raipur districts show a high percentage prevalence of wasting.

**Figure 6:**
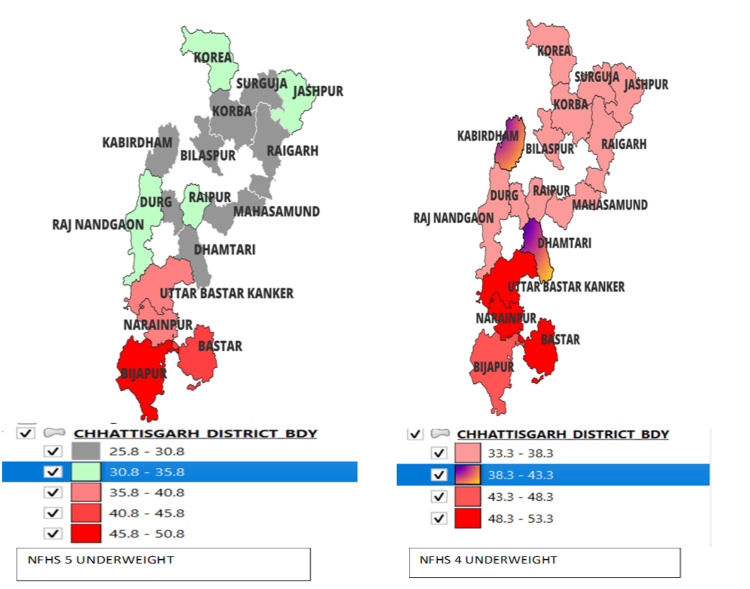
Comparison of underweight under-five children in NFHS-4 and NFHS-5 in the districts of Chhattisgarh. Sources: [16,18]. NFHS, National Family Health Survey

When comparing the percentage prevalence of underweight under-five children in the districts of Chhattisgarh in NFHS-5 and NFHS-4, as Figure 6 suggests, all districts show a decrease in the percentage of underweight except Bijapur. Kabirdham and Kanker districts, on the other hand, exhibit a significant decrease in the percentage of underweight.

Variations in the Relevant Nutritional Determinants

Child malnutrition is caused by immediate factors such as inadequate food, healthcare, and attention to newborns and early children, particularly during the first two years of life. It may be possible to improve nutrition by addressing fundamental and underlying factors, including women's empowerment, food security, hygiene, and interventions such as sanitation programs. Changing the factors that contribute to malnutrition through a range of initiatives and policy tools is necessary to improve nutritional indicators. Changes in the immediate determinants and underlying determinants of nutrition are investigated in this study, along with addressing interventions related to nutrition-specific determinants [14,15].

**Figure 7:**
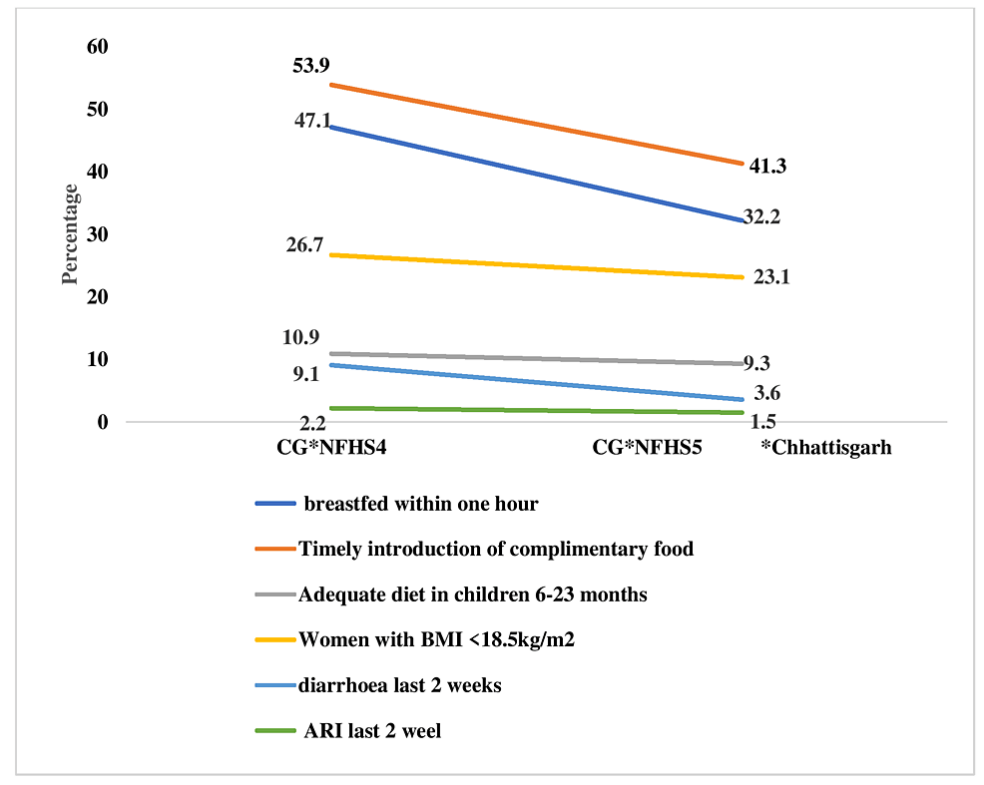
Changes in immediate determinants of nutrition in Chhattisgarh, NFHS-4 and NFHS-5. Sources: [16,18]. NFHS, National Family Health Survey; ARI, acute respiratory infection

Figure 7 shows evidence of the following changes in the immediate determinants of nutrition in Chhattisgarh. In NFHS-5, women with BMI (<18.5 kg/m^2^) constituted a lower percentage (23.1%) compared to NFHS-4 (26.7%). The early initiation of breastfeeding has decreased from 47.1% to 32.2%. It is concerning that the percentage of infants aged 6 months and older who received supplemental feedings has fallen from 53.9% to 41.3%. Only 9.3% of infants aged six to twenty-three months were fed an adequate diet. Acute respiratory infections (ARIs) have decreased slightly from 2.2% to 1.5%, while the percentage of children with diarrhea has decreased from 9.1% to 3.6%.

**Figure 8:**
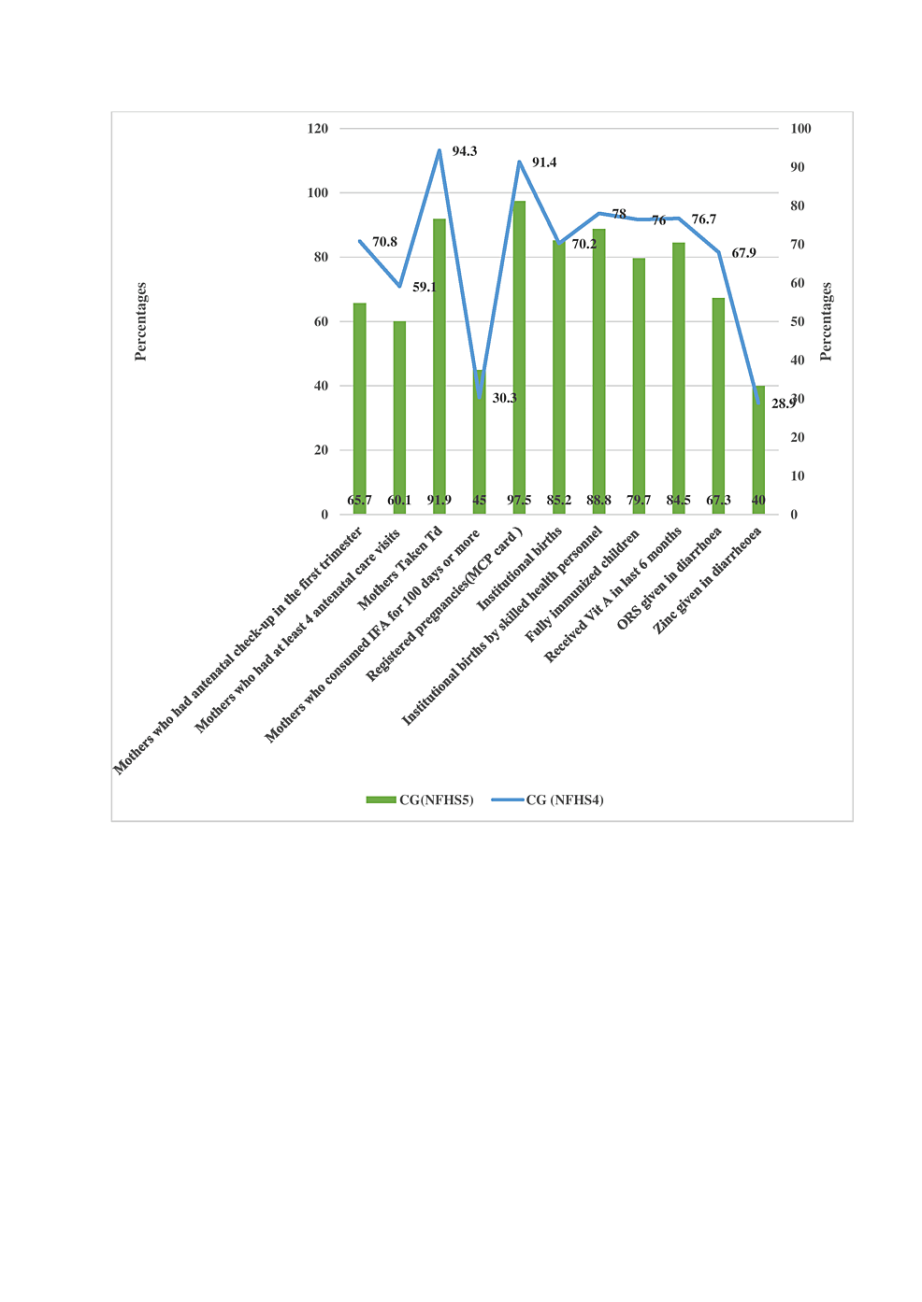
Changes in nutrition-specific interventions in Chhattisgarh (NFHS-and NFHS-5). Sources: [16,18]. NFHS, National Family Health Survey

The coverage of Chhattisgarh's nutrition-specific interventions in NFHS-5 also varies. Figure 8 shows that the percentage of women receiving antenatal care (ANC) in the first trimester of pregnancy dropped from 70.8% to 65.7%, while the percentage of women receiving ANC visits at least marginally improved from 59.1% to 60.1%. Additionally, there was an improvement in the percentage of women giving birth in medical facilities and having professional birth attendants assist them. The use of iron and folic acid (IFA) tablets increased from 30.3% to 45.0% when a woman was pregnant for 100 days or longer. The percentage of children who received vitamin A supplementation increased, and the proportion of fully immunized children also increased from 76.4% to 79.7%. Oral rehydration salts were administered to diarrheal children; the amount decreased slightly from 67.9 to 67.3, but the amount of zinc increased from NFHS-4 to NFHS-5.

**Figure 9:**
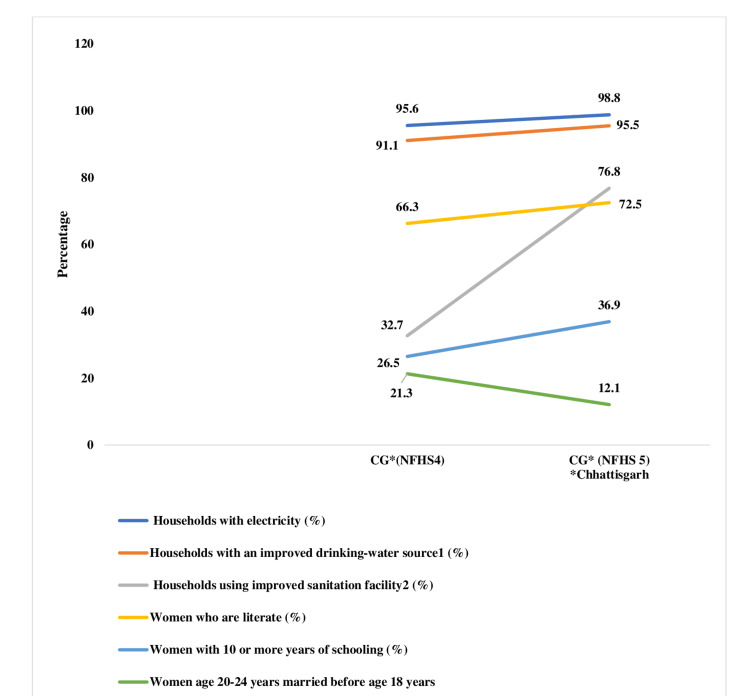
Changes in the underlying determinants of nutrition in Chhattisgarh (NFHS-and NFHS-5). Sources: [16,18]. NFHS, National Family Health Survey

Figure 9 illustrates a significant change in the underlying nutrition determinants across NFHS-5. The percentage of women who have completed 10 or more years of education has increased significantly, but there is still considerable room for improvement among literate women. The percentage of girls married at an early age has decreased from 21.3% to 12.1%. In Chhattisgarh, infrastructure has advanced significantly; over 98% of households now have electricity access, and over 95% have upgraded drinking water sources. The percentage of households utilizing better sanitation facilities has gone from 34.8% to 76.8%, a significant increase. To reach the national mean, gaps between different determinants have been reduced overall and improvements have been made. The criteria that exhibit the least inequalities are the frequency of ARIs, families with electricity and a better source of drinking water, and mothers who obtain mother and child protection cards. Chhattisgarh has performed well when compared to the national average in terms of variables like BMI < 18.5 kg/m^2^, four ANC visits, 100-day IFA tablets, institutional births, and births accompanied by trained healthcare providers. Factors such as early breastfeeding initiation, prenatal checks during the first trimester, 100-day tablets, fully immunized children, literate and educated women, early marriage, and families with improved sanitation vary greatly throughout areas.

Discussion

Variations by state have been observed in nutrition indicators such as underweight, wasting, and stunting in children. While many states and UTs saw improvements, others saw a slight decline in conditions. Stunting and rapid wasting have been noted as improbable drastic changes. One of the main causes of stunting in children is inadequate and inappropriate nutrition in mothers. Between NFHS-4 and NFHS-5, there has been a notable improvement in nutrition-sensitive percentage intervention coverage, such as maternal healthcare and women's empowerment [16,17]. Stunting has decreased, and women's BMI has slightly improved in Chhattisgarh even after these interventions were put into place. The rate of stunting in children varied noticeably between districts [18]. Nine districts had stunting rates that were higher than the 33.33% national average. Sanitation and parental education are the main causes of this disparity. The observed disparities may result from the limited scope of health policies. Stunting could have been influenced by a household's wealth, location, and access to reproductive healthcare (ANC and institutional delivery). The national average for stunted children exceeded in many districts dominated by tribes. Remarkably, the number of stunted, wasting, and underweight children in Chhattisgarh has been declining [18]. Conflicting reports exist regarding the frequency of overweight, wasting, and stunting. It is concerning that being overweight is becoming more common in kids, as it harms their health and has chronic, long-lasting effects that persist into adulthood. Among all the nutritional indicators, the prevalence of anemia is particularly concerning for Chhattisgarh, as it poses a significant public health issue. Chhattisgarh is leading the country in anemia cases among 6 to 59-month-olds, with a notable increase from 58.6% in NFHS-4 to 67.1% in NFHS-5 (a 9.5% increase) [18,19]. In contrast, anemia rose to 52.2% from 50.4% in pregnant women (a 4% increase) and to 57.2% from 53.2% in non-pregnant women (a 1.8% increase). Among the targeted age groups, the prevalence of anemia is over 60% in over three-quarters of Chhattisgarh's districts, suggesting a pattern of anemic individuals experiencing anemia throughout their lives. Similar to this, two-thirds of Chhattisgarh's districts have more than 50% of pregnant women taking IFA tablets experiencing anemia. This indicates that the women of Chhattisgarh may be affected by a different kind of anemia than iron deficiency. Vitamin deficiency anemia and sickle cell anemia, the two common types prevalent in Chhattisgarh state, could be the cause of the high prevalence of anemia in NFHS-5 data. To raise awareness about these forms of anemia and effectively address them, the following steps can be taken: education and screening for these forms of anemia, promotion of a balanced diet rich in iron, vitamin B12, and folate, and timely medical intervention [18]. To improve the indicators, districts with the highest priority need to take immediate action.

Implications and recommendations

A significant portion of India's total malnutrition burden is attributed to Chhattisgarh. Chhattisgarh must immediately set its nutrition target and move quickly to improve all forms of malnutrition in light of India's dedication to global nutrition goals. Numerous nutritional outcomes, most notably anemia, present significant challenges. There is serious concern regarding the sharp rise in anemia among men, women, and children in NFHS-5, which is higher than the national average. The highest cause for concern is the rate of anemia in the entire state of Chhattisgarh. All indicators of anemia prevalence in the state must be promptly and effectively addressed, not just one. The majority of nutrition-specific interventions and underlying determinants have seen improvements in Chhattisgarh since the last NFHS. While Chhattisgarh has not shown significant improvement, even though most dietary indicators have improved, more work has to be done to pay particular attention to the factors that contribute to anemia. A solution to the malnutrition problem in Chhattisgarh must be implemented right away. The state exhibits declining trends in underweight, overweight, severe wasting, stunting, and wasting in children under the age of five. It suggests that Chhattisgarh should implement a robust multipronged strategy that takes into account all types of malnutrition. The state should invest in maintaining adequate intervention delivery during the first thousand days of life when coverage is fairly good, while also focusing on improving the coverage of interventions that lag. Chhattisgarh's immediate determinants like early breastfeeding initiation, complementary feeding techniques, and providing children with a healthy diet must all be prioritized. When it comes to nutrition-specific interventions, we should prioritize improving maternal care during pregnancy, i.e., antenatal check-ups during the first trimester for expectant mothers, with a stronger focus on maintaining the gains made in institutional births and delivery assisted by skilled personnel. Women's education, early marriage, and sanitation are three underlying factors that urgently need to be addressed. Early intervention is necessary to improve women's nutrition, which is crucial for improving pregnancy-related and early child health outcomes. For young girls and soon-to-be mothers, this may have positive long-term effects; iron deficiency is common in the population until that time. Iron supplements and other public health measures are important but insufficient to stop childhood anemia. The best results in raising children's hemoglobin levels may come from a combination of iron supplementation, food fortification initiatives, along with efforts to address maternal anemia, family poverty, and food insecurity. The effectiveness and breadth of the state's program need to be increased. It calls for proactive measures and scaling up of innovations required to address malnutrition and anemia, in addition to stepping up efforts on healthy diets, maternity, infant, and young child nutrition, managing wasting, micronutrient supplementation, school feeding and nutrition, and nutrition surveillance, particularly at inter-district levels. Shortly, advocacy for multisectoral actions is required at all levels and throughout the life course to improve nutrition outcomes. The program's efficiency can be raised by carefully organizing and carrying out the current plans and policies and by promptly monitoring, assessing, and determining whether sufficient funding is available.

Chhattisgarh-specific nutrition initiative

In August 2019, the Chhattisgarh government initiated a program to provide free nutritious food daily to people in the state who are malnourished or anemic [20]. The state government has set a goal to eradicate anemia and malnutrition within the next three years, according to the Chief Minister: "This campaign was launched as a pilot project in July 2019 in the Bastar region and was also carried out in other aspirational districts of the state." A maximum number of prominent charitable organizations, public representatives, NGOs, media groups, and other capable individuals from the districts would be involved in this campaign to eradicate anemia and malnutrition.

Under the Chief Minister’s direction, the state government in Dantewada launched the *Haat Bazaar Health Camp* and the *Suposhan Abhiyan* (nutrition movement) in 2019 as two programs to end child and maternal malnutrition and lower the overall rates of infant and maternal mortality (IMR and MMR). These women, kids, and young girls are receiving a nutritious, nutrient-rich diet as part of a special nutrition program. Their meals include sprouting grains, protein-rich soy and peanuts, eggs for B12, jaggery, green veggies, pickles for addressing iron deficiency, and more. In addition to improving people's health and well-being, the two projects also contribute to their financial well-being. The government is providing jobs for tribal women by employing Anganwadi workers and Self-Help Groups (SHGs) to handle food preparation.

Block ranking exercise (BRE)

An initiative called BRE aims to support the state National Health Mission in strengthening the Anaemia Mukt Bharat program by evaluating its performance at the district and block levels [21]. Nutrition International conducted a BRE pilot project in 12 districts of Chhattisgarh in 2020. A scorecard was made for the intervention districts as part of this initiative. Scores were determined by looking at a few key process indicators, such as coordination committee meetings, reporting, supply availability, and many more, which were connected to the AMB program's output indicators, like the availability of IFA tablets. Additionally, field coordinators received training on how to create monthly district-specific scorecards. The field team used these scorecards in advocacy meetings with district authorities, and nodal officers shared them with them so that corrective actions could be taken within the program. Furthermore, BRE was carried out in the intervention districts to pinpoint problems and find fixes to enhance the way the AMB program was implemented. When compared to non-intervention districts, the coverage of IFA in the intervention districts increased dramatically with the introduction of BRE.

Chhattisgarh (CG) Mukhyamantri Suposhan Abhiyan

The state government has initiated CG Mukhyamantri Suposhan Abhiyan as part of a state-level campaign to combat malnutrition [20]. The following are some of the Abhiyan's salient characteristics and highlights: The CG Mukhyamantri Suposhan Abhiyan, aimed at improving nutrition for girls, women (15-49 years), and children (0-5 years), provides daily wholesome meals. The Chhattisgarh government supplements this with extra eggs, jaggery, and groundnut laddu twice a week to combat anemia and malnutrition. In Bastar, the pilot project of the Harik Naani Bera Campaign benefits around 9,000 mothers and 70,000 children in Anganwadi centers across the state. The Women and Child Welfare Department oversees the Suposhan Abhiyan, providing anthelmintic medications and iron tablets, with a focus on locally sourced, nutrient-dense food. Each day, the CG government distributes free nutritious meals to designated beneficiaries in gram panchayats, including fruits, milk, poultry, soybeans, laddus, bhaji, and other nutrient-rich foods. Governmental and nongovernmental organizations collaborate in this effort, utilizing Anganwadi centers, schools, and other identified distribution points facilitated by the district administration.

Millet’s initiative

To establish Chhattisgarh as the millet hub of India, the state government launched Mission Millet Chhattisgarh in September 2021 [22]. With a focus on 85 blocks in 20 districts and a total budgetary allocation of Rs. 170 crores, its main goal is to promote the cultivation of Kodo millet, little millets, and finger millet throughout the state. An input grant of Rs. 9,000 per hectare has been decided upon. The implementing body for the state's millet procurement and processing was designated as the Chhattisgarh Minor Forest Produce Cooperative Federation. Under the Mukhyamantri Suposhan Yojna, the state government of Chhattisgarh introduced finger millets, or ragi, into the Supplementary Nutrition Program (SNP) in 2018. Under Take Home Ration, pregnant and nursing women, children under the age of six months, severely malnourished children, and girls aged between 11 and 14 receive ready-to-eat food packets containing ragi, wheat, soybean, Bengal gram, sugar, groundnut, and fortified soybean oil.

Kanker

Under this yojna, Kanker initiated the Kanker Kilkari project. Since February 2021, pregnant and nursing women as well as children aged six months to five years have been given ragi halwa and kodo kutki khichadi. The DMF fund of the district provides the funding for this. The Agriculture Department provides these millets (kodo, kutki, and ragi). Kondagaon began the Yojna's kodo and small millets (kutki) khichadi program in May 2021. It is given to malnourished children aged two to six years. The funding for this comes from Women's Self-Help Groups and is provided by the Tribal Department.

Raigarh introduced Ragi on September 15, 2021, in five district blocks. Pregnant women, children aged three to six years, and malnourished children aged six months to three years are all given ragi laddu. Funds from CSR provide the budget for this. Women's Self-Help Groups provide the ragi laddu mix.

## Conclusions

The comparative study concluded that Chhattisgarh should prioritize funding for general nutrition. Infants and young children require extra care to keep them from becoming malnourished. Districts like Janjgir-Chapa, Korba, Raipur, and Durg should be the main focus of Chhattisgarh's attention because they bear a lot of burden. The state should address problems of all possible causes in these districts. The combined effects of anemia and malnutrition may have a direct impact on Chhattisgarh's development. In most nutrition indicators, the state has not made any significant progress. The state government's initiatives are expected to bring about significant changes by reducing malnutrition and anemia rates among children and women, improving overall health outcomes and well-being, enhancing awareness about nutrition, and promoting the consumption of nutritious foods like millet. Therefore, it is imperative that extra care be given to addressing the factors that contribute to anemia and that a plan be put in place to combat malnutrition in Chhattisgarh as soon as possible. It's time to consider nutrition a top priority.

## Appendices

 Appendix A

**Table 1:** Comparison of nutritional status in different districts of Chhattisgarh. BMI, body mass index

		BMI < 25 kg/m^2^		BMI ≥25 kg/m^2^	
Sr No	District Name	NFHS-4	NFHS-5	NFHS-4	NFHS-5
1	Bastar	37.1	34.9	6.3	8.7
2	Bijapur	20.1	43.6	2.4	6
3	Bilaspur	13.5	27.2	10	17.1
4	Dantewada	40.6	25.9	4.9	6.8
5	Dhamtari	29.8	18.4	10	12.7
6	Durg	24.1	12.5	18.6	23.9
7	Janjgir - Champa	27	21.5	13.9	12.8
8	Jashpur	28.3	27.5	8.2	13.4
9	Kabeerdham	32.9	20.6	8.8	11.8
10	Korba	29.9	24.4	14.8	16.2
11	Koriya	24.7	25.1	11.1	17.3
12	Mahasamund	28.6	21.3	7.8	12.6
13	Narayanpur	23.9	28.1	4	5.7
14	Raigarh	28.4	26.9	12.3	14.9
15	Raipur	26.7	14.4	17.1	15.7
16	Rajnandgaon	16.3	25.9	7	12.8
17	Surguja	35.9	26.8	8.6	15.3
18	Uttar Bastar Kanker	35.5	25.5	9.3	6.8

Appendix B

**Table 2:** Prevalence of anemia in different districts of Chhattisgarh in different age groups. NFHS, National Family Health Survey; BMI, body mass index

	Children aged 6 to 59 months (<11 g/dL)		Nonpregnant women (15-49 years) (<12 g/dL)		Pregnant women (15-49) years(<11 g/dL)		All women 15-49 years who are anaemic		All women 15-19 years who are anaemic	
District Name	NFHS-4	NFHS-5	NFHS-4	NFHS-5	NFHS-4	NFHS-5	NFHS-4	NFHS-5	NFHS-4	NFHS-5
Bastar	59.4	80.7	68.2	77.1	57.3	79	67.6	77.2	42.6	80.3
Bijapur	51.3	77.2	70.6	72.9	47.9	60.3	68.7	72.1	77.8	68.7
Bilaspur	29.4	78.1	41.5	59.3	29.8	41.4	40.7	58.5	27.1	63.9
Dantewada	71.3	89.9	75.3	76.7	58.3	63	74.5	76	43.7	75.7
Durg	44.4	57.1	48.9	52	52.6	41.2	49.1	51.7	20	61.8
Janjgir - Champa	35.6	74.1	40.3	66.3	30	60	39.9	66.3	40.8	67.9
Jashpur	31.1	54.1	35.2	61.8	54.5	54.5	35.7	31.5	27	60.3
Kabeerdham	37.6	67.5	34.5	43.9	43	35.6	34.9	43.6	27.5	45.4
Korba	39.1	63.6	45.4	66.9	39.7	54.6	45.1	66.4	36.9	70.5
Koriya	33.7	56.3	36.7	65	34.7	54.1	36.6	64.4	38.8	48.3
Mahasamund	38	75.8	49.4	63.4	53.3	56.5	49.5	63	51	63
Narayanpur	48.2	86.8	59.5	72.8	50.3	57.2	58.9	72	61.7	72
Raigarh	38.8	62.7	41.8	62.5	36.5	54.2	41.6	62.2	36.2	59.5
Raipur	47.1	74.6	50.9	59.8	51	37.1	50.9	59.1	22.2	63.1
Surguja	38.6	51.4	35.1	58.8	37.6	46.4	35.1	58.3	14.7	54.3
Uttar Bastar Kanker	61.9	62.3	67.8	66.3	56.7	33.4	67.5	65.2	67.8	69.2
